# The impact of obesity on the accuracy of DXA BMD for DXA-equivalent BMD estimation

**DOI:** 10.1186/s12891-022-06076-0

**Published:** 2022-12-26

**Authors:** Min-Woo Kim, Dong-Ha Lee, Jung-Wook Huh, Jang-Whan Bai

**Affiliations:** Department of Orthopedic Surgery, Orthopedic Surgeon, Busan Medical Center, Busan, Republic of Korea

**Keywords:** Dual-energy x-ray absorptiometry (DXA), Index of Central Obesity (ICO,), Bone mineral density (BMD), Morphometric texture analysis, Linear regression

## Abstract

**Introduction:**

As the radiomics technique using texture features in CT is adopted for accessing DXA-equivalent bone mineral density (BMD), this study aims to compare BMD by DXA and predicted BMD to investigate the impact of obesity and central obesity in general patients.

**Materials and methods:**

A total of 710 cases (621 patients) obtained from May 6, 2012, to June 30, 2021, were used in the study. We focused both their abdomen & pelvis CT’s first lumbar vertebrae axial cuts to predict estimated BMD and bone mineral content (BMC). In each patient’s CT, we extracted the largest trabecular region of the L1 vertebral body as a region of interest (ROI) using the gray-level co-occurrence matrices (GLCM) technique, and linear regression was applied to predict the indices. Cases were divided by central obesity/overall obesity and normal group by body mass index (BMI), waist circumference (WC), or index of central obesity (ICO) standard.

**Results:**

The coefficients were all above 0.73, respectively. *P*-values from ICO were over 0.05 when the measures were Hip BMD and Hip BMC. In contrast, those from ICO were 0.0131 and 0.0351 when the measures were L1 BMD and L1 BMC, respectively, which show a difference between the two groups.

**Conclusions:**

The CT HU texture analysis method was an effective and economical method for measuring estimated BMD and BMC and evaluating the impact of obesity. We found that central obesity especially exerted an effect on the disturbance of the clinical BMD measurements since groups were significantly different under the ICO standard.

**Supplementary Information:**

The online version contains supplementary material available at 10.1186/s12891-022-06076-0.

## Introduction

Dual X-ray absorptiometry (DXA) is the most popular way of diagnosing osteoporosis [1]. Since DXA takes a two-dimensional measurement of bone mineral density (BMD), it translates as area BMD containing both trabecular and cortical BMD [2]. However, it is presumed that DXA provides inaccurate values due to various types of scanning artifacts [3]. The presence of osteophytes, aortic calcification, and tall patients are some potential factors that might cause higher BMD values. Apart from DXA, one of the most common tools for measuring BMD is quantitative computed tomography (QCT). QCT measures trabecular volumetric bone density (vBMD) and the result is valued in milligrams per cubic centimeter of calcium hydroxyapatite. The vBMD is not affected by the bone volume, osteophytes of cortical bone, or circumjacent soft tissues such as fat tissue [4].

The concept of the BMD and T-score values from QCT are commonly lower than those from DXA were demonstrated by many studies [5–8], while others found that they are similar [9, 10]. DXA measurement itself and hypothetically inhomogeneity of fat distribution seem to be the main reason for showing errors in relatively high clinical BMD [11]. Other studies demonstrated that DXA precision errors positively correlate with increasing Body Mass Index (BMI) [6] and surrounding adipose tissues in the spine [7].

These findings imply that obese patients can be classified as normal BMD in DXA scans but with abnormal BMD and osteoporosis in QCT scans, thus DXA can fail to initiate therapy when it is needed. Also, changes in body compound and fat layers distribution might influence common osteoporosis status assessed by DXA in postmenopausal females. Although fat layer distributions influence clinical BMD precision, it is still showing no clarity whether central obesity or overall obesity affects the scanning of true bone density.

Although BMI is used commonly for measuring obesity, it does not take into consideration of the fat layer distribution which is important in scanning artifacts of DXA [12]. Waist circumference (WC; > 88 cm) and waist-to-height ratio (WHtR; > 0.5) are easy and convenient measures for central obesity in a public health context [13]. As the BMD is usually measured at the lumbar spine, these measures might be useful in the interpretation of lumbar spine BMD taken by DXA, but the evidence is short.

Recently, a radiomics technique using CT texture features was adopted to access estimated BMD [14–16]. Unlike QCT, CT cannot analytically compute the quantity due to the use of single energy, thus it needs special post-processes using CT images in Hounsfield units (HU) to predict BMD. The processes manually segment a bone area of interest, extract the features using gray-level co-occurrence matrices (GLCM) [17, 18], and optimally weigh the features using simple machine-learning techniques [19].

In this study, we take advantage of the estimation method to indirectly identify that obesity influences the clinical BMD. One presumption is that the features extracted from the only bone areas in CT are rarely dependent on obesity like QCT. Then, if obesity causes the discrepancy between real BMD and clinical BMD, it will bring different statistics of estimation errors between the obesity group and the normal group. BMI and WC and the Index of Central Obesity (ICO) are adopted to investigate the impact of central obesity on the BMD estimation.

## Materials and methods

### Subject enrollment and allocation

**Fig. 1:**
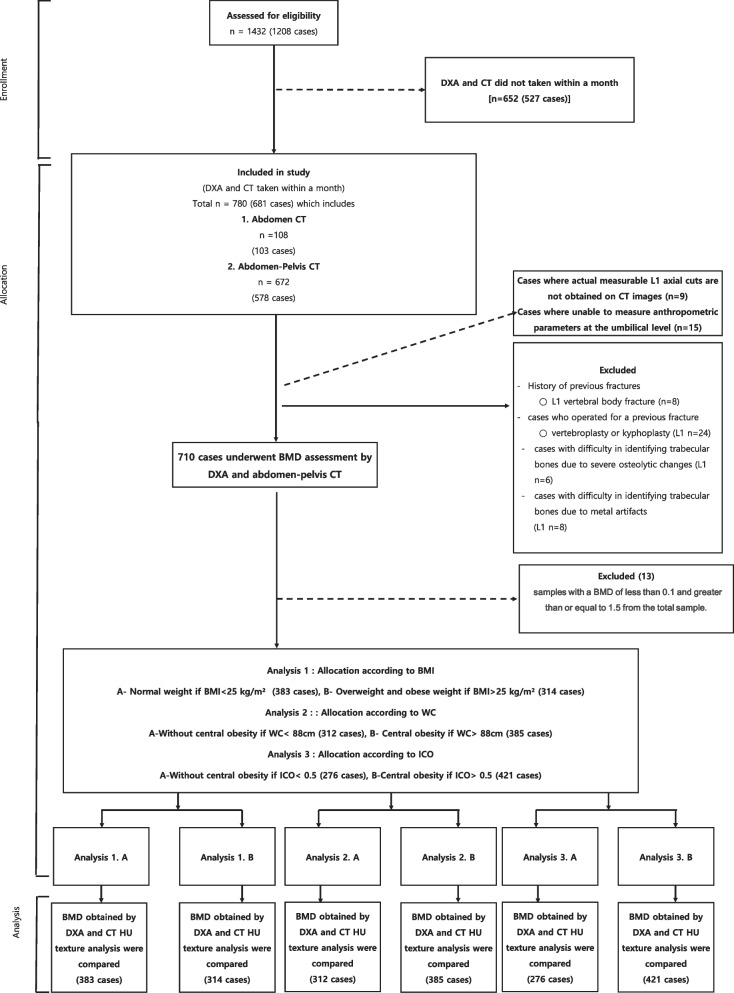
Conceptual diagram showing workflow and stratification of cases

The Institutional Review Board "Public Institutional Review Board Designated by Ministry of Health and Welfare" (P01-202,109–21-014) approved this study. A total of 1423 cases (1208 patients) who had undergone both CT and DXA in a single institution between May 6, 2012, to June 30, 2021, were initially corrected. Among them, we selected 780 cases (681 patients) with (1) abdominal CT or abdomen-pelvis CT with a complete first lumbar vertebra (L1) axial cut, (2) abdominal CT or abdomen-pelvis CT completely showing umbilical cord axial cut, and (3) less than a one-month gap between CT and DXA scan dates. Next, we excluded 70 cases (60 patients) with (1) a history of previous L1 vertebral body fracture, (2a) history of cement or metal artifacts of a previous fracture and refracture, and (3) difficulty in identifying trabecular bones. Thus, 710 cases (612 patients) were selected as shown in Fig. 1.

Additionally, we considered clinical BMD conditions that the samples whose BMD is less than 0.1 g/cm^2^ and greater than 1.5 g/cm^2^ were excluded (13 patients) as outliers. The samples were finally divided by normal and overweight/obese under the BMI, WC, or ICO standard as shown in Fig. 1.

### Anthropometric measurements

A seca digital scale was used to measure the patient’s height and weight. Body mass index (BMI) was calculated as weight (kg) divided by the square height expressed in meters. BMI values in the range 19—25 kg/m2 were considered normal weight, while subjects with BMI ≥ 25 kg/m2 and ≥ 30 kg/m2 were the cut-off levels for overweight and obese subjects, respectively. Waist Circumference was measured at the abdominal-pelvis CT’s axial cut where the umbilical cord was visible along the axis of the lumbar vertebrae and cut-off > 88 cm was used as an indicator of central obesity. Index of central obesity (ICO) is a term defining the ratio of WC and height. The International Diabetes Federation (IDF) suggested that it better predict central obesity where ICO > 0.5- central obesity and ICO < 0.5—no central obesity.

### Imaging protocols for CT and DXA

A Siemens (SOMATOM 128, Definition AS +) scanner (Siemens Healthcare, Forchheim, Germany) was used for CT scans, for every scan, the protocol was a single-energy CT with 120 kVp, 247 mA, dose modulation 0.6-mm collimation. The effective pitch was 0.8 and the reconstruction kernel was B60 (sharp). Reconstructed slice thicknesses were set at 5.0 mm and 3.0 mm for chest CT and lumbar spine CT, respectively. And the slice increments for abdomen and pelvis CT were 5.0 mm and 3.0 mm, respectively. For DXA scans, a standard device with a standard protocol (GE Lunar Prodigy, GE Healthcare) was used and reports were obtained using vendor-specific software (Physicians Report Writer DX, Hologic, Discovery Wi, USA).

### Region of interest

**Fig. 2:**
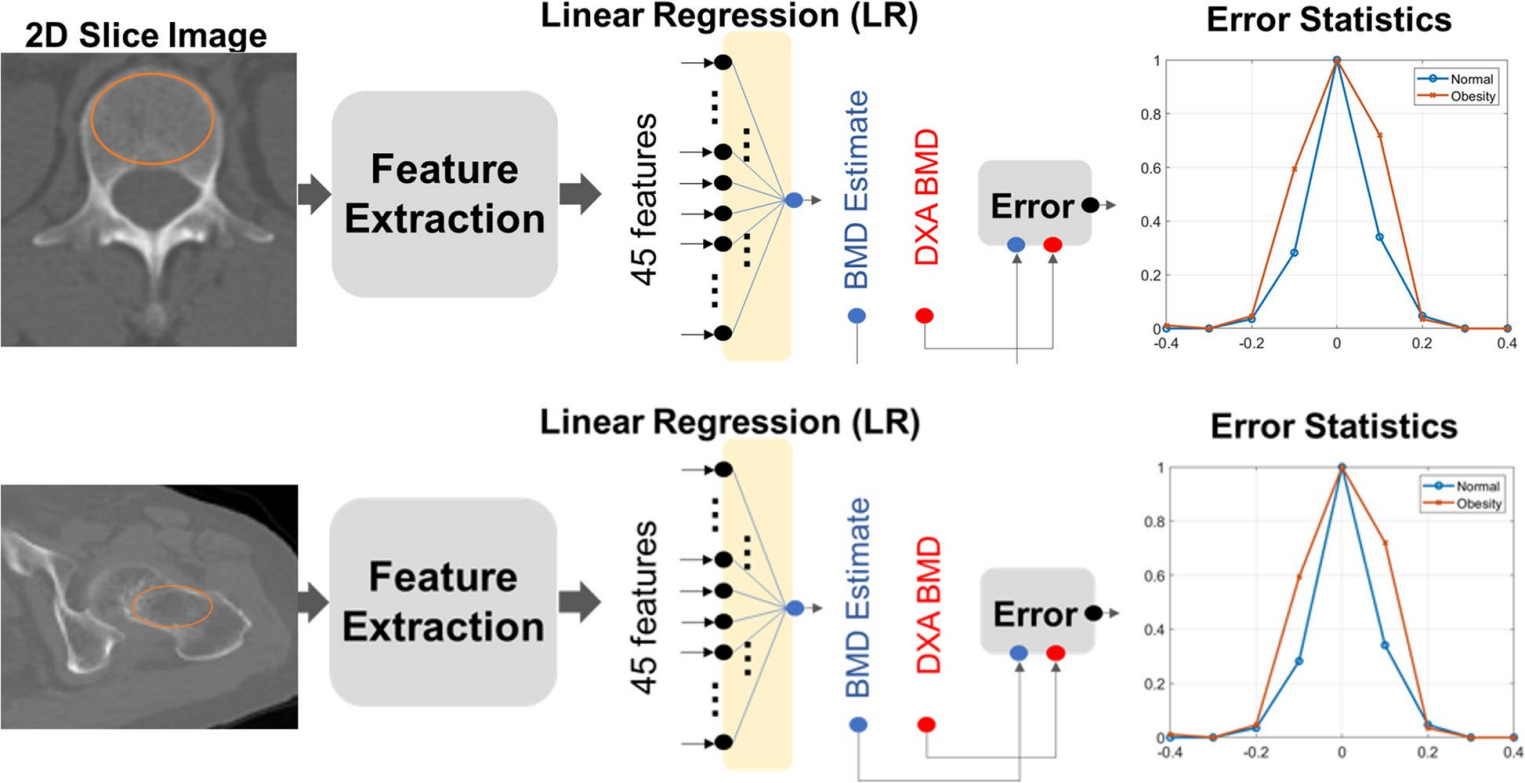
Schematic flow for BMD estimation from computed tomography BMD, bone mineral density

Regions of interest (ROIs) for statistical measurement from the CT axial cuts of every patient, we selected one slice image such that it contains the maximum axial trabecular area of the bone. Of the many methods available for isolating ROIs, the thresholding method was chosen for this study. A 2-dimensional (2D) slice image was chosen from the CT axial cut of every patient wherein the 2D image contained the maximum axial trabecular area of the L1 spine body or the femoral neck. As shown in Fig. 2, we conducted texture analysis in a circular region covering most of the trabecular area.

### Clinical BMD estimation using CT

Figure 2 illustrates the schematic flow of the BMD estimation. A total of 45 features were extracted from the ROIs, of which five features were intensity-based and extracted using a histogram, and 40 texture-based features were extracted using a GLCM matrix. One estimation clinical BMD was then computed from the features using conventional linear regression (LR).

### Statistical analysis

Statistical analyses were performed using MATLAB 9.10 R2021a (MathWorks, Natick, Massachusetts, USA). The paired t-test was used to verify differences in clinical results and patient demographics between the two groups. Power analysis revealed an effect size of 0.5, the statistical significance of 0.05, and the statistical power of 0.90 for both groups. Mean absolute error was used to compare p-values between the two groups.

## Results

### Patient characteristics

A total of 697 cases (334 males and 363 females) were included in the final analysis. The normal group’s cases were 383 (207 males and 176 females) and the overweight/obese group’s cases were 314 (127 males and 187 females). Their mean age was 54.13 ± 11.64 years, 53.49 ± 9.61 years and 54.62 ± 10.24 years, respectively. The mean gap between CT and DXA was 1.76 ± 6.89 days. The patient demographics for each group are summarized in Table 1.

**Table 1:** Demographics for the Total, Normal and Overweight/Obese groups

	**Total**	**Normal**	**Overweight/ Obese**	***P*-value of T-test total and normal**	***P*-value of T-test normal and overweight/obese**	***P*-value of T-test total and overweight/obese**
Cases (n)	**697**	**383**	**314**	**-**	**-**	**-**
Age (y)	**54.13 ± 11.64**	**53.49 ± 9.61**	**54.62 ± 10.24**	**0.21**	**0.13**	**0.24**
Sex (male/female)	**334/363**	**207/176**	**127/187**	**0.23**	**0.19**	**0.27**
BMI (kg/m2)	**24.66 ± 2.3**	**21.82 ± 1.8**	**27.71 ± 1.9**	**0.11**	**0.09**	**0.14**
WC (cm)	**87.32 ± 9.6**	**84.54 ± 6.7**	**89.55 ± 7.1**	**0.12**	**0.10**	**0.14**
ICO	**0.52 ± 0.09**	**0.51 ± 0.07**	**0.53 ± 0.09**	**0.16**	**0.12**	**0.19**
Gap between CT and DXA dates (days)	**1.76 ± 6.89**	**1.69 ± 4.89**	**1.81 ± 5.12**	**0.42**	**0.31**	**0.46**

The general obesity rate was a total of 316 which is 44.5% of total cases. The central obesity rate was 424 (59.7%). Table 2 shows the percentages of cases by 3 different criteria for each group.

**Table 2:** Percentages of cases and *p*-values of cases allocation according to BMI, WC, ICO criteria

Analysis 1 A.—Normal (%)	383 (54.9%)		
Analysis 1 B.—Overweight cases (%)	**302 (43.3%)**		
Analysis 1 B.—Obese cases (%)	**12 (1.7%)**		
Analysis 2 A.—Cases with WC < 88 cm (%)	**312 (44.8%)**		
Analysis 2 B.—Cases with WC > 88 cm (%)	**385 (48.5%)**		
Analysis 3 A.—Cases with ICO < 0.5 (%)	**276 (39.6%)**		
Analysis 3 B.—Cases with ICO > 0.5 (%)	**421 (59.7%)**		
*P*-value of T-Test Analysis 1 A. – 2 A. (Age, Sex, BMI)	**0.45**	**0.39**	**0.38**
*P*-value of T-Test Analysis 1 A. – 3 A. (Age, Sex, BMI)	**0.12**	**0.16**	**0.13**
*P*-value of T-Test Analysis 2 A. – 3 A. (Age, Sex, BMI)	**0.25**	**0.28**	**0.35**
*P*-value of T-Test Analysis 1 B. – 2 B. (Age, Sex, BMI)	**0.35**	**0.22**	**0.32**
*P*-value of T-Test Analysis 2 B. – 3 B. (Age, Sex, BMI)	**0.11**	**0.15**	**0.09**
*P*-value of T-Test Analysis 2 B. – 3 B. (Age, Sex, BMI)	**0.21**	**0.15**	**0.11**

### Correlation test

**Fig. 3:**
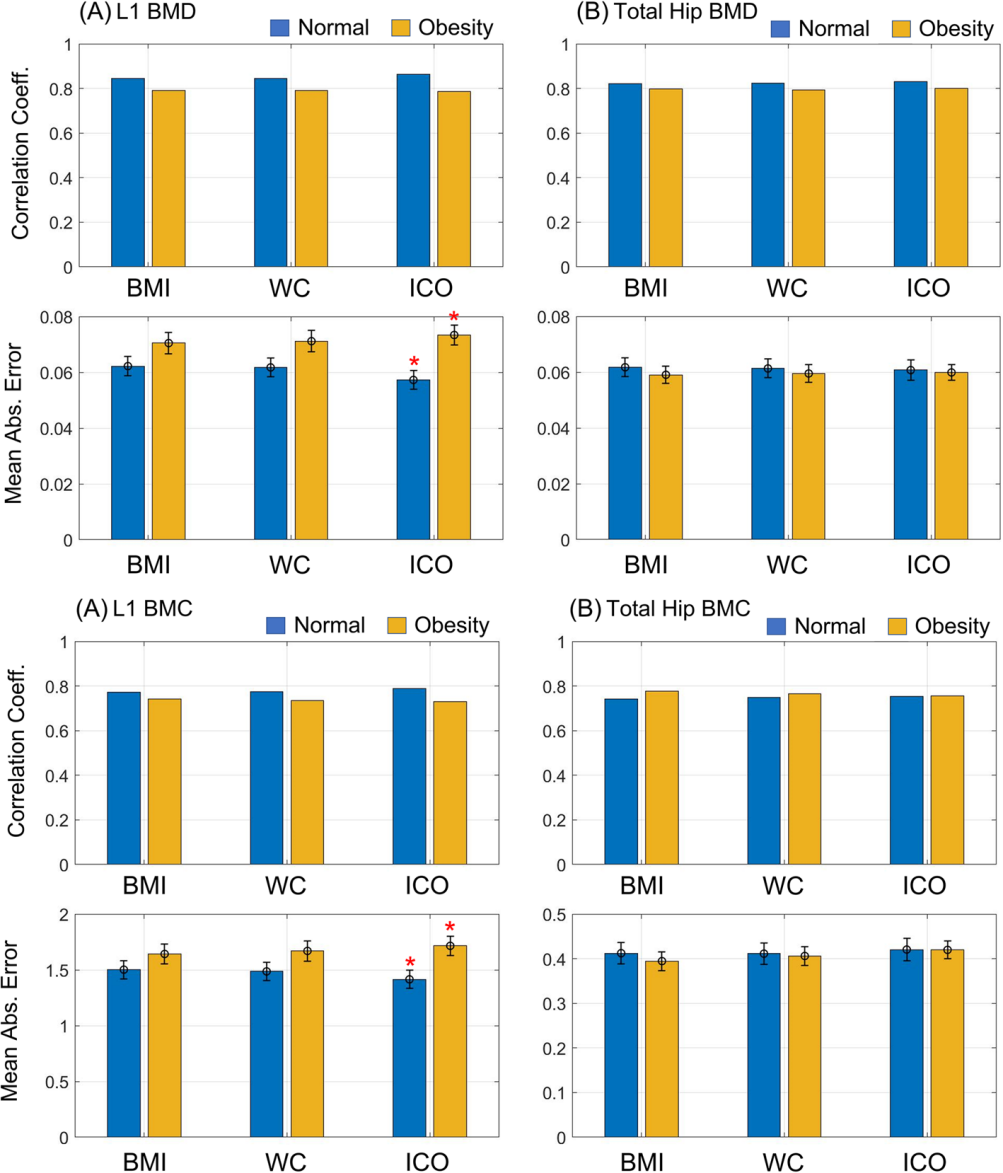
1) Correlation coefficient and Mean Absolute Error with (A) L1 BMD, (B) Total Hip BMD 2) Correlation coefficient and Mean Absolute Error with (A) L1 BMC, (B) Total Hip BMC

Table 2 summarizes the correlation coefficient and mean absolute error (MAE) between the estimated BMD and clinical BMD (g/cm2) values. We applied the LR model to 24 datasets and the ANN model to one dataset. The histograms in Fig. 3 show the clinical BMD (g/cm2) reference values and the corresponding estimated values across cases. The plot indicates the estimate values for the test samples, which were predicted by the network after being trained by the training samples. When the LR model was applied to datasets 1, 2, 3, 4.0.24, the coefficients were all above 0.65, respectively. Especially, when comparing between normal ICO and ICO > 0.5 groups, the coefficient values are significantly different, and that central obesity has a high contribution to clinical BMD measurement error (Table 3).

**Table 3:** Spearman correlation coefficients and mean absolute errors between bone mineral density values from DXA and CT HU texture analysis according to with central obesity / without central obesity

Correlation Coefficient (Mean absolute error)			DXA based clinical BMD (g/cm2)			
			**L1 BMD (g/cm2)**	**Total Hip BMD (g/cm2)**	**L1 BMC (g)**	**Total Hip BMC (g)**
**L1 estimated BMD by CT HU texture analysis**	**BMI (kg/m2)**	**Analysis 1 A.—Normal weight if < 25 kg/m**^**2**^	**0.7900 (0.0548)**	**0.7539 (0.0642)**	**0.7651 (1.5325)**	**0.7354 (0.4231)**
		**Analysis 1 B.—Overweight and obese weight if > 25 kg/m**^**2**^	**0.6657 (0.0625)**	**0.7712 (0.0573)**	**0.742 (1.6444)**	**0.7768 (0.3967)**
	**WC (cm)**	**Analysis 2 A.—Without central obesity if < 88 cm**	**0.7849 (0.0555)**	**0.7558 (0.0635)**	**0.7662 (1.5220)**	**0.7419 (0.4204)**
		**Analysis 2 B.—central obesity > 88 cm**	**0.6542 (0.0644)**	**0.7666 (0.0577)**	**0.7351 (1.6715)**	**0.7639 (0.4075)**
	**ICO**	**Analysis 3 A.—Without central obesity if < 0.5**	**0.8125 (0.0517)**	**0.7488 (0.0659)**	**0.7796 (1.4526)**	**0.7418 (0.4295)**
		**Analysis 3 B.—Central obesity if > 0.5**	**0.6594 (0.0658)**	**0.7843 (0.0576)**	**0.7303 (1.7153)**	**0.7545 (0.4210)**

In the normal ICO group, the L1 BMD mean absolute error values ​​were 0.0573 and the L1 BMC mean absolute error values ​​were 1.4152, and in the ICO > 0.5 group, the values ​​were 0.0734 and 1.7153, respectively. Based on the L1 BMD mean absolute error value, the p-value between normal ICO and ICO > 0.5 groups showed a statistically significant difference of 0.0019 and based on the L1 BMC mean absolute error value, the p-value was also statistically significant at 0.0160. There was no statistically significant difference in the other groups.

WC (cm) /ICO is a measure of central obesity while BMI (kg/m2) is an index of overall obesity. As shown in Fig. 3, the difference between the normal WC cm group / normal ICO group and the WC > 88 cm / ICO > 0.5 groups was more distinct than normal BMI (kg/m2) and the overweight/obese BMI (kg/m2) groups. Although the normal WC cm / WC > 88 cm groups and normal BMI (kg/m2) vs overweight/obese BMI (kg/m2) groups did not show differences, the p-values between the two groups using the L1 BMD mean absolute error were 0.1162 (. normal WC cm / WC > 88 cm groups) and 0.1697 (. normal BMI (kg/m2) vs overweight/obese BMI (kg/m2)), and the L1 BMC mean absolute error was 0.2220 (. normal WC cm / WC > 88 cm), 0.3542 (. normal BMI (kg/m2) vs overweight/obese BMI (kg/m2)) showed a tendency to show a difference. In contrast, p-values from the. normal ICO and ICO > 0.5 groups were 0.0131 and 0.0351 when the measures were L1 BMD (g/cm2) and L1 BMC (g), respectively.

## Discussion

In this study, a texture-based technique in CT was conducted to create a linear regression model to confirm the error and correlation between DXA measures and DXA-equivalent estimates. We analyzed them in L1 and hip cases for normal and obesity groups divided by either BMI (kg/m2), WC (cm), or ICO. Since all cases provided a high correlation (0.73–0.87), the estimation proved an effective method for accessing DXA BMD (g/cm2) using only CT. In addition, we found that the degree of central obesity causes the noise in DXA measurement by identifying the statistics of estimation errors between two groups.

Our results are stand-in with the findings that the main reasons for the influence on the precision errors of the spine may be the greater thickness of the spine and the greater inhomogeneity of the soft tissues of the abdomen [20]. Also, the location of soft tissues in the abdomen varies, which can be a source of errors. Therefore, as central obesity increases, the BMD difference is likely to increase due to soft tissue unevenness between scans.

Contrary to the clinical L1 BMD (g/cm2) and BMC (g) results, the two groups did not show differences when the measurements occurred in the hip area. Considering the hip anatomy, when DXA x-rays are transmitted through the lumbar spine side, there are almost no large structures that can cause noise such as abdominal fat (thickness of sagittal adipose tissue) and abdominal aortic calcification. Since the femoral artery passing through the hip also passes to the medial side rather than the femur, there is no overlap of bone and artery [21]. In a study to measure the changes in DXA based BMD values by placing exogenous fat on the thighs or abdomen in subjects with different bodies, it was found that fat placement did not affect the BMD of the proximal femur, but rather the BMD values of the lumbar spine lead to measurement errors at this specific site of the human body [22]. Authors suggested that the discordance between correlation results at total femur and L1 of those two formulas might be due to the possibility that imaging artifacts including anatomy of the region of the aimed bone might adversely affect two imaging methods. Also, current bone mass measurement techniques show limitations in clinically overweight/obese patients and those with dramatic weight loss or gain.

Our results suggest that the bone density results of DXA in overweight/obese patients should be interpreted cautiously. Also, in our study, DXA BMD and DXA-equivalent estimation of L1 correlated positively with measures of overall obesity, while those were less correlated with measures of central obesity Suggesting that increasing overall obesity might cause less noises of BMD values by DXA estimated BMD. Estimation using CT is less likely to be affected by surrounding adipose layers and soft tissue inhomogeneity. The higher percentage body fat groups showed increased precision errors of the spine. It is associated with increased tissue thickness and fat inhomogeneity. The precision errors that increase with increasing body fat %, especially in the lumbar spine than in the total hip region, within this study fit this effect well. As Fig. 3 shows the results of Mean Absolute Error’s difference are more pronounced in the L1 spine than in the total Hip [23].

The reason why CT was selected for texture analysis is as follows. Abdomen CT or abdomen and pelvis CT are often taken for health checkups, and DXA for diagnosing osteoporosis was taken at the same time. Abdomen or abdomen-pelvis CT includes ROI (L1) that scans DXA for BMD measurement, and CT suitable for measuring ICO WC such as including axial cuts of the linea alba. CT HU measurement can simply represent BMD using tissue density of vertebrae trabecular bone mass. Calculation of GLCM as a secondary texture measurement is one of the widely used methods in texture analysis. By extracting several statistical parameters from the GLCM, it is possible to quantify the spatial relationship between pixels within the area to be investigated. These include energy, contrast, entropy, autocorrelation, correlation, inverse moment, cluster shade, and shading studied to verify texture properties [24]. Whitmarsh et al. developed DXA software-computing structural parameters for the proximal femur by extending it from a 2D model to a 3D model and compared them to QCT with accuracy [25]. We believe that our study shows high value in that CT is general-purpose and viewable from a variety of perspectives. Additionally, CT allows for the screening of patients with osteoporosis risk without additional diagnostic tests.

There are several limitations of our study. First, since our study was limited in sample size and obtained from a primarily Asian cohort from a single center, further studies with a bigger sample size are needed to verify the findings. Second, our results using CT HU of L1 were only compared with L1 DXA BMD and not compared with those of other lumbar vertebrae. BMD (g/cm2) and BMC (g) correlation studies with other lumbar vertebrae except for L1 on abdomen-pelvis CT are necessary for the future. Third, the fact that comparative analysis was not conducted by separately examining the presence or absence of aortic calcification was insufficient to find the exact cause in interpreting the results. Finally, the cross-sectional form of the study has limitations in evaluating whether estimated BMD estimation using CT is superior to DXA in the prediction of future fracture risk in overweight/obese compared to normal-weight patients.

## Conclusion

The CT HU texture analysis method was an effective and economical method for measuring estimated BMD and BMC and evaluating the impact of obesity. We found that central obesity especially exerted an effect on the disturbance of the clinical BMD measurements since groups were significantly different under the ICO standard.

## Supplementary Information

**Additional file 1.** 

**Additional file 2.**

## Data Availability

Applicable. All data generated or analyzed during this study are included in this published article [and its supplementary information files].
