# Supplementary material for: The impact of obesity on the accuracy of DXA BMD for DXA-equivalent BMD estimation
Source: BMC Musculoskelet Disord. 2022 Dec 26;23:1130. doi: 10.1186/s12891-022-06076-0 (PMC9791746; doi:10.1186/s12891-022-06076-0)
Supplement: Supplementary file 1 — Additional file 1. [file 12891_2022_6076_MOESM1_ESM.docx]

**Appendix for more interested readers**

DXA BMD estimation using CT

Fig.3. illustrates the schematic flow of the BMD estimation. Specifically, from the CT axial cuts of every patient, we selected one slice image such that it contains the maximum axial trabecular area of the bone. A total of 45 feature values were obtained from every area where the 45 features are based on an intensity histogram (of CT HU) and the others are based on GLCM, one of the texture analysis techniques. A total of 45 features were extracted from the ROIs, of which five features were intensity-based and extracted using a histogram, and 40 texture-based features were extracted using a GLCM matrix. One estimation DXA BMD was then computed from the features using conventional linear regression (LR).

Our key assumption is that the estimation error is contributed by obesity. In specific, the LR model can be represented by $y_{DXA}+e_{1}=y_{CT}+e_{2}$ where a ${ya}_{DXA}$ denotes the DXA BMD and $y_{CT}$ denotes the estimate from a a CT HU. The error $e_{1}$ is to discrepancy between DXA BMD and the real BMD and the error $e_{2}$ is discrepancy between BMD estimate and real BMD. Our hypothesis is that the two errors are mostly independent, and $e_{1}$ is strongly influenced by obesity in contrast with $e_{2}$ since DXA covers body beyond the bone. The regression finds the estimate $y_{CT}$ from 45 features such that the variance, $var\left( e_{1}-e_{2} \right)=var\left( e_{1} \right)+var(e_{2})$, is minimized. Thus, the obesity group might have higher estimation error than normal group due to the higher variance $v\left( e_{1} \right)$. `

They were input into a linear regression (LR) model and an artificial neural network (ANN) model. The LR model estimated BMD through a linear combination of the 45 input values. The ANN model was a fully connected neural network wherein the first, second, and third hidden layers had eight, eight, and two nodes, respectively. Additionally, every node had a non-linear operator (rectified linear unit) (Fig. 2)
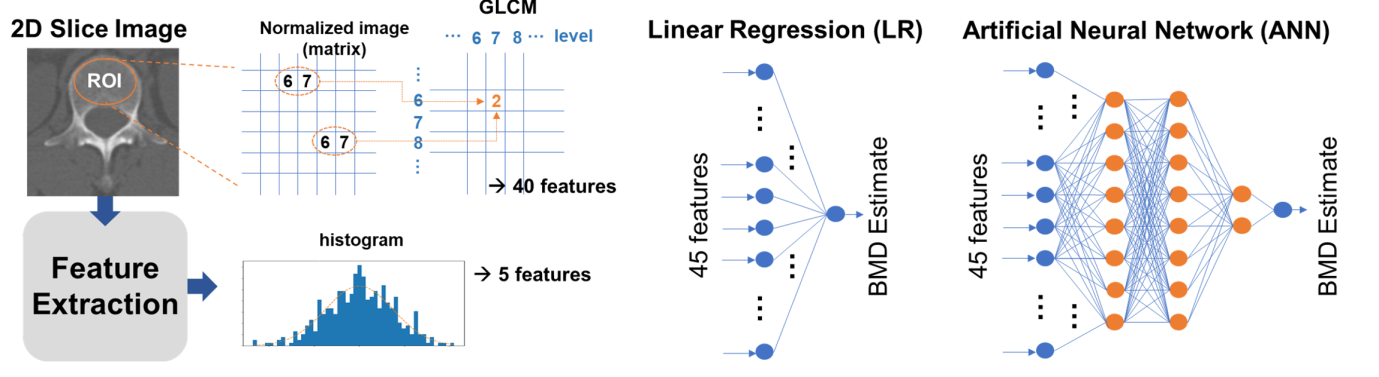
.

Intensity-based features were obtained using an ROI image histogram. They include mean, standard deviation, skewness, kurtosis, and entropy. These basic numerical values reflect properties of bone intensities, including brightness, asymmetry, randomness, uniformity, and sharpness. In addition, 40 features were extracted from the texture analysis to obtain information about the spatial relationships between adjacent pixels across a 2D image. The features were derived from the GLCM (Fig. 2). Provided that options are N gray levels and horizontal direction, the matrix size is N×N where the (i,j)th element corresponds to the total number of instances in which two horizontally adjacent pixels have values i and j in the discretized and normalized ROI image with intensity scale ranging from 1 to N. In this study, we created eight GLCMs for every sample ROI image using four levels (N=16, 32, 64, 128) and two directions (horizontal and vertical), and measured five statistics (entropy, contrast, correlation, homogeneity, and variance) for each GLCM.

BMC is widely used as the most important material for bone fragility, strength, and structure; therefore, it plays an important role in predicting fractures. Since the trabecular bone has a high bone turnover rate, it is a bone structure that reflects bone evaluation the most by responding sensitively to metabolic stimuli. BMC covering trabecular bone that carries high potential accuracy of indicator of osteoporosis and fracture risk. In addition, Matkovic et al found that true bone density does not provide accurate bone status concerning growth because the bone is a metabolically active organ.

(Matkovic V, Jelic T, Wardlaw G, et al. Timing of peak bone mass in Caucasian females and its implication for the prevention of osteoporosis. Inference from a cross-sectional model. The Journal of clinical investigation. 1994;93(2):799-808.)
